# Statistical Modeling and Prediction for Tourism Economy Using Dendritic Neural Network

**DOI:** 10.1155/2017/7436948

**Published:** 2017-01-26

**Authors:** Ying Yu, Yirui Wang, Shangce Gao, Zheng Tang

**Affiliations:** Faculty of Engineering, University of Toyama, Toyama-shi 930-8555, Japan

## Abstract

With the impact of global internationalization, tourism economy has also been a rapid development. The increasing interest aroused by more advanced forecasting methods leads us to innovate forecasting methods. In this paper, the seasonal trend autoregressive integrated moving averages with dendritic neural network model (SA-D model) is proposed to perform the tourism demand forecasting. First, we use the seasonal trend autoregressive integrated moving averages model (SARIMA model) to exclude the long-term linear trend and then train the residual data by the dendritic neural network model and make a short-term prediction. As the result showed in this paper, the SA-D model can achieve considerably better predictive performances. In order to demonstrate the effectiveness of the SA-D model, we also use the data that other authors used in the other models and compare the results. It also proved that the SA-D model achieved good predictive performances in terms of the normalized mean square error, absolute percentage of error, and correlation coefficient.

## 1. Introduction and Literature Review

With the impact of global internationalization, tourism is also in a state of rapid development. As we all know, tourism's impact on the economic and social development of a country can be enormous. It can not only up business, trade, and capital investment but also create jobs and entrepreneurialism for workforce and protect heritage and cultural values (as shown in Table 1). Each country wants to know the data of its inbound visitors and tourism in order to choose an appropriate strategy for its economic well-being. Hence, a reliable forecast is needed and plays a major role in tourism planning.

Accurate forecasts build the foundation for better tourism planning and administration. Then more efficient forecasting techniques in tourism demand studies are being called for.

Over the past two decades, tourism demand modeling and forecasting which are two of the most important areas in tourism research have attracted more and more attention of both academics and practitioners. As Song and Li concluded, twenty years ago, there were only a handful of academic journals that published tourism-related research [1]. Now there are more than 70 journals that serve a thriving research community covering more than 3000 tertiary institutions across five continents. However, there has not been a panacea for tourism demand forecasting.

In recent years, statistics has been widely applied to the tourism economy under study. Among the statistical methods, time series forecasting is an important area of forecasting. And it can be classified into two categories: the linear methods and the nonlinear methods. The most popular of the linear methods are the Naïve model [2–5], the exponential smoothing (ES) model [2, 6], and the autoregressive integrated moving averages (ARIMA) model [3, 4, 6]. Among them, the most advanced forecasting model of linear methods is autoregressive integrated moving average model (ARIMA) which has been successfully tested in many practical applications. If the linear models can approximate the underlying data generating process well, they could be considered as the preferred models. However, if the linear models fail to perform well in both in-sample fitting and out-of-sample forecasting, more complex nonlinear models should be considered. Based on this view, many scholars have also turned to nonlinear methods such as the neural network (NN) [3, 4, 7, 8]. Although there are still a few doubts about neural network based tourism demand forecasting, it is generally believed that the nonlinear methods outperform the linear methods in modeling the economic behavior and efficiently helping wise decision-making.

Neuron networks have been regarded by many experts as a promising technology for time series forecasting. Consequently, in the last few decades, more than 2000 articles on neural network forecasting have been published covering a wide range of applications [9]. Compared to statistical forecasting techniques, neural network approaches have several unique characteristics, such as (1) being both nonlinear and data driven, (2) having no requirement for an explicit underlying model, and (3) being more flexible and universal and thus applicable to more complicated models [10]. Furthermore, Nelson et al. and Zhang and Kline [11, 12] suggested that time series preprocessing (e.g., detrending and deseasonalizing) contributes significantly to neuron network model performance.

Up to now, there are many researchers using a lot of methods to forecast the tourism demand. And they can be divided into three types: time series, neural network, and combined models. In 2014, Teixeira and Fernandes published [13], in which the three methods are all mentioned. Except those, there are also a lot of authors using the three methods separately. For example, Box et al., Cho, Chu, Song, and Li, Law, Qu, and Zhang, Shahrabi et al., Li et al., and Kawakubo and Kubokawa have used the traditional time series methods to forecast the tourism demand [1, 3–7, 14–17]. As neural network is widely known, there are many authors turning to use the neural network to forecast the time series data such as Chen et al., Claveria and Torra, Davies et al., Constantino et al., Law, Lin et al., and Pai and Hong [3, 4, 8, 18–22]. With the progress of science, more and more methods are being used. The combined models are the most popular methods in them. And, up to now, Bates and Granger, Chen, Shen et al., and Yan have used this method and got the expected results [23–26]. Besides these, some other methods such as support vector regression [27, 28] and novel hybrid system [29, 30] are proposed. They have made great achievements in the optimization problem and the prediction problem; however, the data preprocessing and the late parameter selection problem are relatively complex.

When analyzing time series data, we should pay particular attention to the seasonality of the time series involved. Seasonality is a notable characteristic of tourism demand and cannot be ignored in the modeling process when monthly data are used. How to handle the seasonal fluctuations of tourism data has always been an important issue in tourism demand forecasting. We always use normal quantile transform or seasonal difference method to eliminate the impact of seasonality [31, 32].

In this paper, we mix the most advanced linear model (SARIMA model) with the innovative neural network model (DNN model) together and call the mixed model SA-D model. We obtained that the SA-D model performs much better than the DNN model in the tourism demand forecasting as the comparing results showed.

This paper is organized as follows. In Section 2, the SARIMA model, the DNN model, and the combined model (SA-D model) are described.  Section 3 describes the data set and discusses the evaluation methods to compare the forecasting methods and takes statistical tests to check the SA-D model and then compares the models that other authors had given by using the same data. After that, the experimental results are given.  Section 4 provides concluding remarks.

## 2. Modeling (Statistical Modeling and Neural Network)

A time series model explains a variable with regard to its own past and a random disturbance term. Time series models have been widely used for tourism demand prediction in the past four decades. In this section, two models are described as follows.

### 2.1. ARIMA Model and SARIMA Model

ARIMA is the most popular linear model for forecasting time series. It has made great success in both academic research and industrial applications. A general ARIMA model is ordered by (*p*, *d*, *q*), and it can be written as

(1) $$\begin{array}{c} \phi \left(\;B\;\right)\nabla^{d}x_{t}=\theta \left(\;B\;\right)\varepsilon_{t}, \end{array}$$

where *x*_*t*_ and *ε*_*t*_ represent the number of visitors and random error terms at time *t*, respectively. *B* is a backward shift operator defined by *Bx*_*t*_ = *x*_*t*−1_ and related to ∇ by ∇ = 1 − *B*; ∇^*d*^ = (1 − *B*)^*d*^; *d* is the order of differencing. *ϕ*(*B*) and *θ*(*B*) are autoregressive (AR) and moving averages (MA) operators of orders *p* and *q*, respectively, and they are defined as

(2) ϕB=1−ϕ1B−ϕ2B2−⋯−ϕpBp,θB=1−θ1B−θ2B2−⋯−θqBq.

*ϕ*_1_, *ϕ*_2_,…, *ϕ*_*p*_ are the autoregressive coefficients and *θ*_1_, *θ*_2_,…, *θ*_*q*_ are the moving average coefficients.

When fitting ARIMA model to the raw data, the ARIMA model involves the following four steps:

- Identification of the ARIMA (*p*, *q*, *d*) structure
- Estimation of the unknown parameters
- Goodness-of-fit tests on the estimated residuals
- Forecast future outcomes based on the known data

*ε*_*t*_ should be independently and identically distributed as normal random variables with mean = 0 and constant variance = *σ*^2^. The roots of *ϕ*_*p*_(*x*_*t*_) = 0 and *θ*_*q*_(*x*_*t*_) = 0 should all lie outside the unit circle. It was suggested by Box et al. that at least 50 or preferably 100 observations should be used for the ARIMA model [14].

If the data has significant seasonal changes periodically. We can use the SARIMA model which uses the seasonal difference method to eliminate the effects of seasonal cycles. However, if the seasonality is regarded as deterministic, introducing seasonal dummies into the time series models would be sufficient in accounting for the seasonal variation. To test for the presence of seasonal unit roots, the HEGY test [33] is widely used. Unlike the HEGY test, an alternative method known as the test for fractional integration to test the seasonal components in the time series was introduced in 2004 [34]. Another approach to model seasonal fluctuations is to use the periodic autoregressive model. This model allows parameters to vary according to the seasons of a year and therefore may reflect seasonal economic decision-making more adequately than constant parameter specifications.

### 2.2. DNN Model (Neuron Model with Dendritic Nonlinearity)

Recently, more and more nonlinear forecasting models are proposed to address the time series' issues. As Song and Li concluded, among them, ANNs (artificial neural networks) are receiving increasing interests due to their ability to imperfect data, functions of self-organizing, self-study, data-driven, associated memory, and arbiter function mapping [1].

As we all know, the structure of every neuron is unique; it contains three parts: the cell body, dendrite, and axon. The dendrite receives the signal from other neurons; then the signal is computed at the synapse and transmitted to the cell body. If the signal into the cell body exceeds the holding threshold, the cell will fire and send the signal down to other neurons through axon.

**Figure 1:**
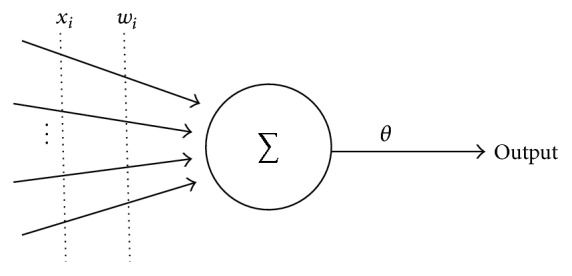
McCulloch-Pitts model.

In 1943, a simple neuron model is proposed by McCulloch and Pitts in which the dendrites and synapses are independents and there are no effects on them from one to another (Figure 1) [35]. However, in 1987, Minsky and Papert indicated that the McCulloch-Pitts model is limited to solving complex problems [36].

Different from the McCulloch-Pitts model which does not consider the dendritic structure in the neuron, neuron model with dendritic nonlinearity model (DNN model) is proposed in our researches. The DNN model can be generalized as follows:

1. The dendrites can be initialized by any arbitrary decision.
2. The synapses on the same branch interact with each other.
3. The nonlinear interaction produced in a dendrite can be expressed by a logical network.
4. After learning, the branches' ripened number and the locations and types of synapses on the branches will be synthesized.

**Figure 2:**
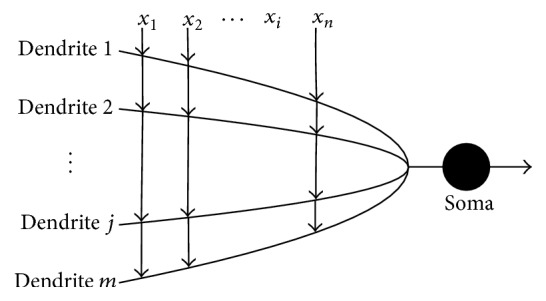
Neuron model with dendritic nonlinearity.

As shown in Figure 2, the dendritic branches receive signals from *x*_1_ to *x*_*n*_ and then perform a simple multiplication on their own signal. At the junction of the branches, the outputs are summed up and then conducted to soma (the cell body). If the input of the soma exceeds a threshold, the cell will fire it and send it to other neurons through the axon.

*Synaptic Function*. In the connection layer, a sigmoid function reflects the interaction among the synapses in a dendrite. The output of the synapse whose address is from the *i*th (*i* = 1,2,…, *m*) input to the *j*th (*j* = 1,2,…, *n*) branch is given by the following equation:

(3) $$\begin{array}{c} Y_{ij}=\frac{1}{1+e^{-k\left(w_{ij}-\theta_{ij}\right)}}. \end{array}$$

*w*_*ij*_ and *θ*_*ij*_, respectively, mean the connection parameters, and *k* is a positive constant. When *k* becomes large enough, the sigmoid function will turn out to be similar to a step function. Through the change of the value of *w*_*ij*_ and *θ*_*ij*_, four types of synaptic connections can be defined: a direct connection, an inverted connection, a constant-0 connection, and constant-1 connection. 

*Dendritic Function*. It performs a simple multiplication on various synaptic connections of the branch. The output of the *j*th branch is given by

(4) $$\begin{array}{c} Z_{j}=\overset{n}{\underset{i=1}{\prod }}Y_{ij}. \end{array}$$

*Membrane Function*. It is approximated as follows:

(5) $$\begin{array}{c} V=\overset{m}{\underset{j=1}{\sum }}Z_{j}. \end{array}$$

*Soma Function*. The function of the soma is described by a sigmoid operation; when *k* is taken as a positive constant, *γ* is taken as a threshold from 0 to 1.

(6) $$\begin{array}{c} O=\frac{1}{1+e^{-k\left(V-\gamma \right)}}. \end{array}$$

*Learning Function*. Because DNN is a feed-forward network with continuous functions, the error back-propagation-like algorithm is valid for DNN. By using the learning rule, the error between the target vector and the actual output vector can be expressed as follows:

(7) $$\begin{array}{c} E=\frac{1}{2}{\left(\;T-O\;\right)}^{2}. \end{array}$$

And, according to the gradient descent learning algorithm, the synaptic parameters *w*_*ij*_ and *θ*_*ij*_ can be modified in the direction to decrease the value of *E*. The equations are shown as follows:

(8) Δwijt=−μ∂E∂wij,Δθijt=−μ∂E∂θij,

where *μ* is a positive constant that represents the learning rate. A low learning rate makes the convergence very slow, while a high learning rate is difficult for making the error converge. And the partial differentials of *E* with respect to *w*_*ij*_ and *θ*_*ij*_ are computed as follows:

(9) ∂E∂wij=∂E∂O·∂O∂V·∂V∂Zj·∂Zj∂Yij·∂Yij∂wij,∂E∂θij=∂E∂O·∂O∂V·∂V∂Zj·∂Zj∂Yij·∂Yij∂θij.

### 2.3. The Combined Model (SA-D Model)

Both linear and nonlinear models have achieved successes in their own linear or nonlinear problems. However, none of them is a universal model that is suitable for all situations. Bates and Granger said that a combined model having both linear and nonlinear modeling abilities will be a good alternative for forecasting the time series data [23]. Both the linear and nonlinear models have different unique strength to capture data characteristics in linear or nonlinear domains, so the combined model proposed in this study is composed of the linear component and the nonlinear component. Therefore, the combined model can model linear and nonlinear patterns with improved overall forecasting performance.

It may be reasonable to consider a time series to be composed of a linear autocorrelation structure and a nonlinear component which can be performed as

(10) $$\begin{array}{c} Y_{t}=L_{t}+N_{t}\left(\;10\;\right), \end{array}$$

where *L*_*t*_ is the linear component and *N*_*t*_ is the nonlinear component of the combined model. Both *L*_*t*_ and *N*_*t*_ have to be estimated for the data set. First, the author let linear model (here we use the SARIMA model to perform the obvious seasonal trends) to model the linear part; then the residuals from the linear model will contain only the nonlinear relationship. Let *R*_*t*_ represent the residual at time *t*; then we can know

(11) $$\begin{array}{c} R_{t}=Z_{t}-\hat{L_{t}}\left(\;11\;\right), \end{array}$$

where $\hat{L_{t}}$ denotes the forecast value of the linear model at time *t*. By modeling residuals using nonlinear model (here we use the DNN model), nonlinear relationships can be discovered. In this paper, we built the model with the following input layers:

(12) $$\begin{array}{c} R_{t}^{linear}=f^{nonlinear}\left(\;R_{t-1}^{linear},R_{t-2}^{linear},R_{t-3}^{linear},R_{t-4}^{linear}\;\right)+e_{t}, \end{array}$$

where *R*_*t*_^linear^ represents the residual at time *t* from the ARIMA model, *f*^nonlinear^ is a nonlinear function determined by the DNN model, and *e*_*t*_ is the random error. And the combined forecast can be performed as

(13) $$\begin{array}{c} \hat{Y_{t}}=\hat{L_{t}}+\hat{N_{t}}\left(\;13\;\right), \end{array}$$

where $\hat{N_{t}}$ is the forecast value of (12).

## 3. Results and Prediction

### 3.1. Data Set and the Process

**Figure 3:**
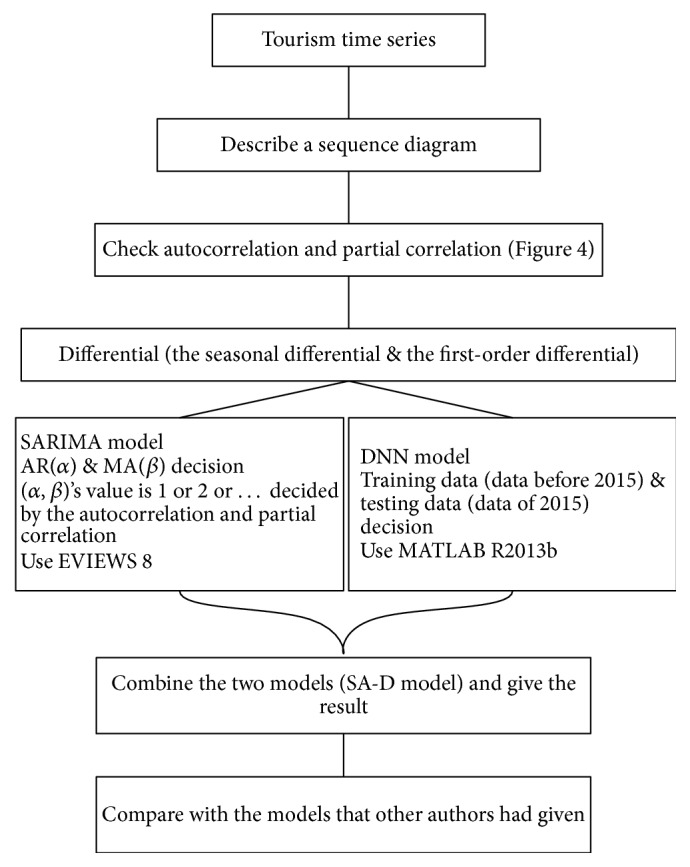
Process of data set.

Due to rapid economic growth and international tourism promotion, the number of tourists coming to Japan is greatly increasing year by year. Here we choose the inbound tourists from 2009:1 to 2015:12. And the process of data set is shown in Figure 3. The collected data were divided into two sets: the training data (data before 2015) and the testing data (data of 2015) [37, 38].

### 3.2. Evaluation Methods

Some quantitative statistical metrics such as normalized mean square error (NMSE), absolute percentage of error (APE), *R* (correlation coefficient), and program running time (PRT) are used to evaluate the forecasting performance of the forecasting models (Table 2). NMSE and APE are used to measure the deviation between the predicted and actual values. The smaller the values of NMSE and APE are, the closer the predicted values to the actual values are. The metric* R* is adopted to measure the correlation of the actual and the predicted values. The PRT can measure the running speed of the models.

### 3.3. Experimental Results

**Figure 4:**
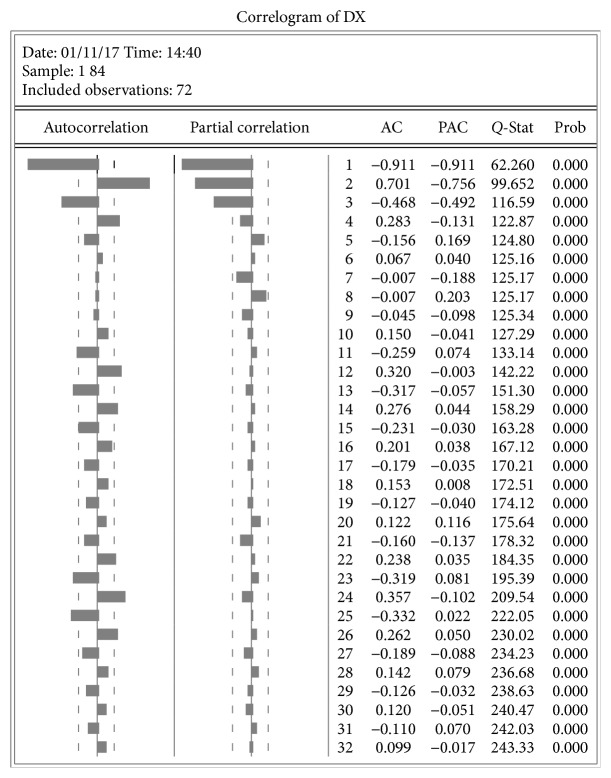
Autocorrelation and partial correlation.

For the data having significant seasonal changes periodically, we use the SARIMA model in this paper to eliminate the linear trend. As Figure 4 shows, we can decide the possible generations of the ARIMA model and use the Akaike Information Criterion (AIC) to test which of the generations is the best.

Through the SARIMA model, we get the data that has no linear trend and train the data separately by the DNN model and the SA-D model. We can get the results of the DNN model and the SA-D model as follows.

**Figure 5:**
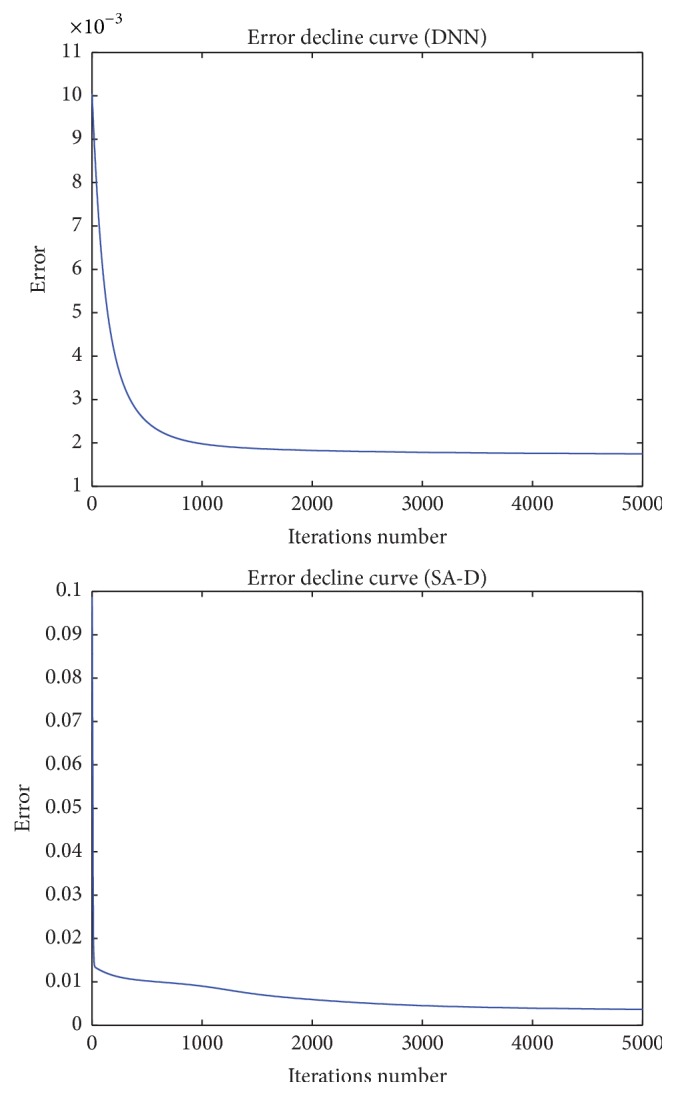
Error decline curve of the DNN model and the SA-D model.

**Figure 6:**
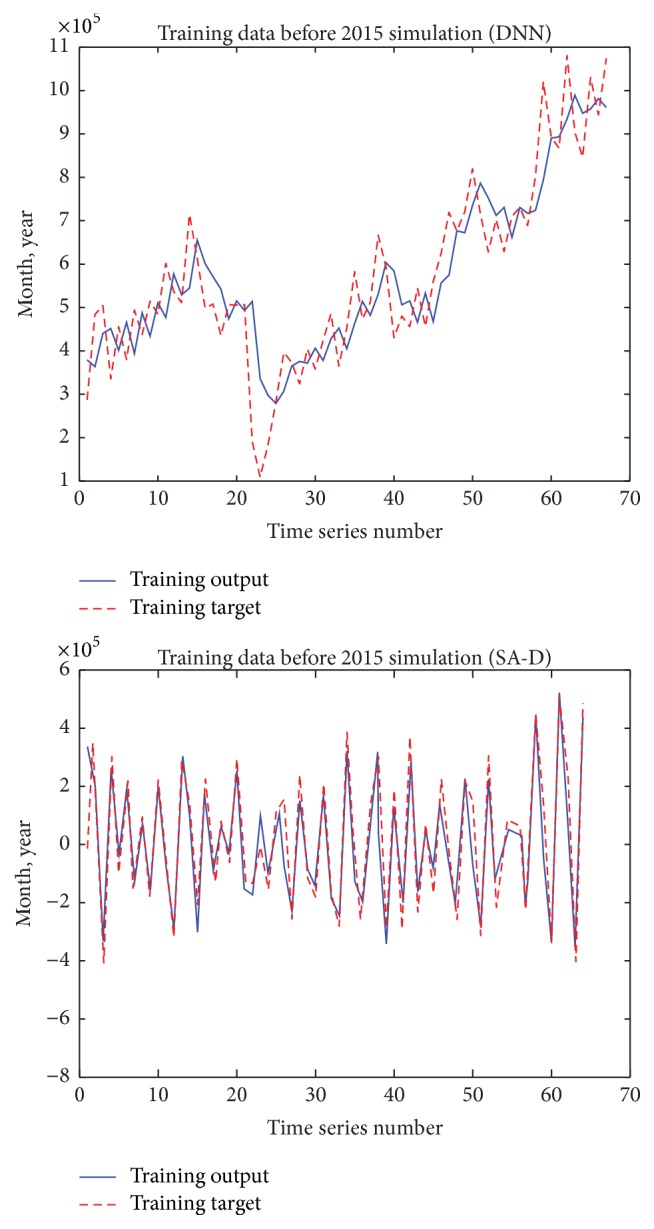
Training data before 2015 simulation of the DNN model and the SA-D model.

**Figure 7:**
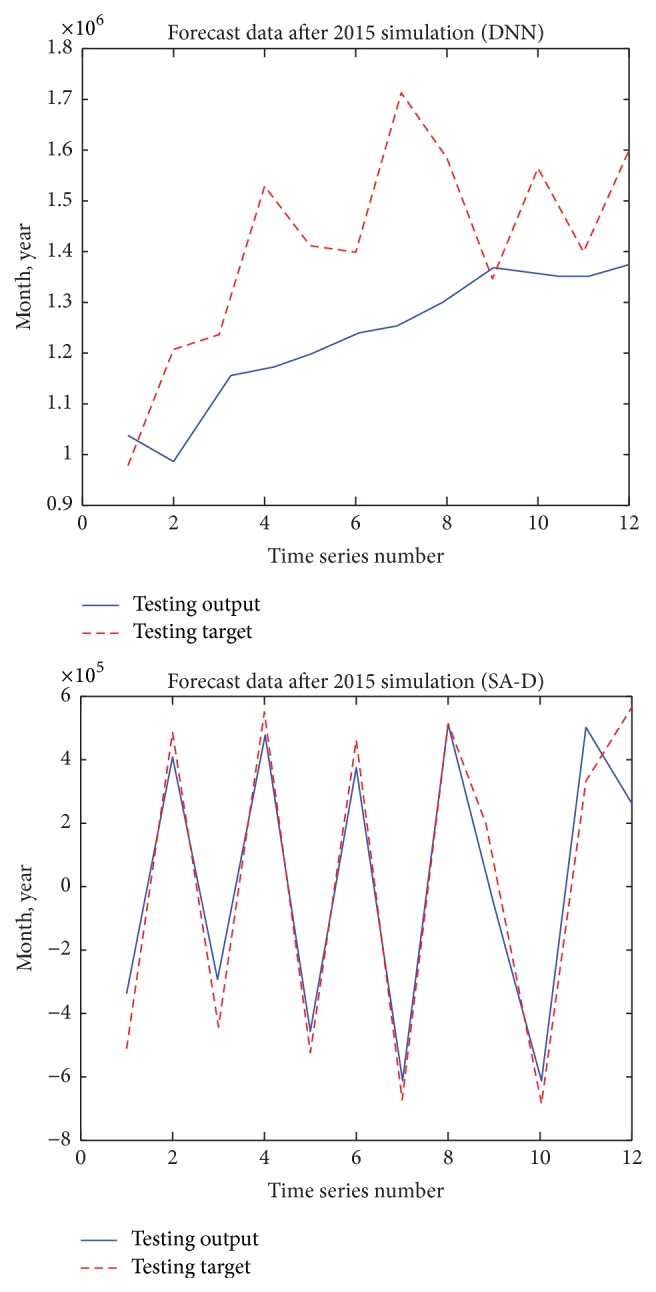
Forecast data after 2015 simulation of the DNN model and the SA-D model.

As Figures 5–7 show, we can see that the results of the SA-D model perform much better than those of the DNN model. In order to deeply evaluate the performance of the DNN model and the SA-D model, we calculate APE, NMSE, and* R* of the testing data set as Table 3 shows.

We can see that although the PRT of the DNN model is rapider than that of the SA-D model, the NMSE, APE, and* R* of the SA-D model are much better than those of the DNN model.

### 3.4. Models Comparison

To demonstrate the validity of the SA-D model, we train the same data that other authors had used in the other combination models and compare the results of the SA-D model and the other combination models. We collected the monthly outbound traveling population data of Taiwan to three areas (Americas, Europe and Oceania) from the Tourism Bureau, M.O.T.C. Republic of China (Taiwan). The study time ranges from January of 1998 to June of 2009 [39]. The collected data were divided into two parts, training data (data from 1998 to 2007) and testing data (data after 2007), for each tourism demand time series. The author scaled the data within the range of (0,1) through the following formula:

(14) $$\begin{array}{c} \frac{x_{t}-x_{min}}{x_{max}-x_{min}}\times 0.7+0.15. \end{array}$$

So we use the data with the same preset as the author did and without the data preset separately and get our experimental results.

Before comparing with the models, we summarize the experimental results based on the orthogonal array, factor assignment, and statistical tests as Table 4 shows. Here the MSD values are calculated by $\bar{x}\pm s$, where $\bar{x}$ means the mean of the results over 20 runs and *s* means the standard deviation. It can verify whether the data is closer to reality or not. And *p* value can determine whether the residual is white noise sequence or not after the statistical test by using QLB statistic. Finally, we choose the result of number 7 to do the comparison.

As Table 5 shows, our model had much better results than other authors' models. But we have to say that the data preset by (14) made the results better and reduced the running time of program.

## 4. Conclusions

In this study, we proposed a new model, the SA-D model, which mixed the SARIMA model and the DNN model together. First, we used the data collected from Japan Tourism Agency Ministry of Land, Infrastructure, Transport and Tourism and Japan National Tourism Organization to compare the SA-D model and DNN model; the results showed that the SA-D model performed much better in fitting and forecasting the time series data. Then we verified the effectiveness of our model by comparing with other authors' models and got the expected result.

The contributions of this study lie in two aspects. Our study is based on neuron model with dendritic nonlinearity model and it theoretically strengthens the assumption that a neural network model performs better than linear models when forecasting nonlinear variables.

This study which mixed the linear model and the nonlinear model together opens the door for further combination models with different methods and models.

## Figures and Tables

**Table 1:** Inbound tourism consumption.

Products	(Billion Yen)		
	Same-day visitors	Tourists	Total visitors
Characteristic products	0	1167	1167
Accommodation services	0	496	496
Food and beverage servicing services	0	303	303
Passenger transport services	0	328	328
Travel agency, tour operator, and tourist guide services	0	8	8
Cultural services	0	10	10
Recreation and other entertainment services	0	8	8
Miscellaneous tourism service	0	14	14
Connected products	0	483	483

Total	0	1650	1650

**Table 2:** Calculations of the performance metrics.

Metrics	Calculation
NMSE	NMSE = $\frac{\sum_{i=1}^{n}(a_{i}-b_{i}{)}^{2}}{n\sigma^{2}}$;
	$\sigma =\frac{\sum_{i=1}^{n}(a_{i}-\overset{-}{a}{)}^{2}}{n-1}$
APE	APE = $\frac{\sum_{i=1}^{n}|(a_{i}-b_{i})/a_{i}|}{n}\times 100\%$
*R*	*R* = $\frac{\sum_{i=1}^{n}a_{i}b_{i}}{\sqrt{\sum_{i=1}^{n}a_{i}^{2}}\times \sqrt{\sum_{i=1}^{n}b_{i}^{2}}}$
PRT	Decided by the actual operation

Note: *a*_*i*_ and *b*_*i*_ are the actual values and the predicted values.

**Table 3:** The compared results of the DNN model and the SA-D model.

Metrics	The DNN model	The SA-D model
NMSE	2.245	0.219
APE	0.87	0.78
*R*	0.32	0.89
PRT	The DNN model is rapider than the SA-D model	

**Table 4:** Results based on the orthogonal array factor assignment and statistical tests of the SA-D model.

Number	*M*	*μ*	*k*_soma_	*θ*_soma_	MSD	*p*
1	15	0.05	1	0	0.401 ± 0.169	0.1938
2	15	0.05	3	0.3	0.386 ± 0.170	0.2013
3	15	0.01	5	0.5	0.391 ± 0.171	0.1854
4	15	0.01	10	0.9	0.389 ± 0.167	0.191
5	25	0.05	1	0	0.392 ± 0.165	0.2563
6	25	0.05	3	0.3	0.395 ± 0.164	0.2742
7	25	0.01	5	0.5	0.398 ± 0.161	0.3011
8	25	0.01	10	0	0.390 ± 0.168	0.2916
9	30	0.05	1	0.9	0.402 ± 0.172	0.1928
10	30	0.05	3	0.3	0.399 ± 0.171	0.1897
11	30	0.01	5	0.5	0.394 ± 0.168	0.2001
12	30	0.01	10	0.9	0.397 ± 0.170	0.1936

Note: *M* means number of dendrites.

**Table 5:** Comparison of the SA-D model and the other combination models.

	Americas	Europe	Oceania
ARIMA + BPNN			
APE	13.41	12.95	13.46
NMSE	0.3992	0.8153	0.5327
*R*	0.9918	0.9917	0.9856
ARIMA + SVR			
APE	11.46	11.37	11.87
NMSE	0.2878	0.6316	0.5102
*R*	0.9923	0.9917	0.9871
The SA-D model (with data preset as other authors did)			
APE	9.61	9.73	9.89
NMSE	0.2788	0.4561	0.4968
*R*	0.9934	0.9921	0.9864
The SA-D model (without data preset)			
APE	10.34	10.51	10.87
NMSE	0.3458	0.5619	0.6027
*R*	0.9912	0.9906	0.9891
